# Neuronal and Synaptic Plasticity in the Visual Thalamus in Mouse Models of Glaucoma

**DOI:** 10.3389/fncel.2020.626056

**Published:** 2021-01-15

**Authors:** Matthew J. Van Hook, Corrine Monaco, Elizabeth R. Bierlein, Jennie C. Smith

**Affiliations:** ^1^Department of Ophthalmology and Visual Sciences, Truhlsen Eye Institute, University of Nebraska Medical Center, Omaha, NE, United States; ^2^Department of Cellular and Integrative Physiology, University of Nebraska Medical Center, Omaha, NE, United States; ^3^Department of Obstetrics and Gynecology, Olson Center for Women’s Health, University of Nebraska Medical Center, Omaha, NE, United States; ^4^Department of Pharmacology and Experimental Neuroscience, University of Nebraska Medical Center, Omaha, NE, United States

**Keywords:** glaucoma, lateral geniculate nucleus, thalamus, DBA/2J mouse, microbead occlusion model, ocular hypertension, intrinsic excitability, synaptic transmission

## Abstract

Homeostatic plasticity plays important role in regulating synaptic and intrinsic neuronal function to stabilize output following perturbations to circuit activity. In glaucoma, a neurodegenerative disease of the visual system commonly associated with elevated intraocular pressure (IOP), the early disease is associated with altered synaptic inputs to retinal ganglion cells (RGCs), changes in RGC intrinsic excitability, and deficits in optic nerve transport and energy metabolism. These early functional changes can precede RGC degeneration and are likely to alter RGC outputs to their target structures in the brain and thereby trigger homeostatic changes in synaptic and neuronal properties in those brain regions. In this study, we sought to determine whether and how neuronal and synaptic function is altered in the dorsal lateral geniculate nucleus (dLGN), an important RGC projection target in the thalamus, and how functional changes related to IOP. We accomplished this using patch-clamp recordings from thalamocortical (TC) relay neurons in the dLGN in two established mouse models of glaucoma—the DBA/2J (D2) genetic mouse model and an inducible glaucoma model with intracameral microbead injections to elevate IOP. We found that the intrinsic excitability of TC neurons was enhanced in D2 mice and these functional changes were mirrored in recordings of TC neurons from microbead-injected mice. Notably, many neuronal properties were correlated with IOP in older D2 mice, when IOP rises. The frequency of miniature excitatory synaptic currents (mEPSCs) was reduced in 9-month-old D2 mice, and vGlut2 staining of RGC synaptic terminals was reduced in an IOP-dependent manner. These data suggest that glaucoma-associated changes to neuronal excitability and synaptic inputs in the dLGN might represent a combination of both stabilizing/homeostatic plasticity and pathological dysfunction.

## Introduction

Changes in synaptic and neuronal function are hallmarks of numerous neurological and neurodegenerative diseases (Wishart et al., 2006; Hall et al., 2015; Wang et al., 2016, 2017; Bae and Kim, 2017; Kim et al., 2017). In many cases, these are likely to be the result of early-stage homeostatic plasticity, which modulates neuronal response properties to maintain firing rate and compensate for altered circuit activity (Turrigiano, 2012; Wondolowski and Dickman, 2013). Such homeostatic modulation is accomplished by scaling presynaptic neurotransmitter release mechanisms, alterations in the balance of excitation and inhibition, modulation of postsynaptic neurotransmitter receptor complement and function, and tuning of intrinsic neuronal excitability and spiking behavior. In cases of disease or injury, homeostasis can make up for circuit dysfunction, but only to a point, after which disease processes are likely to overwhelm homeostasis and trigger unchecked dysfunction and degeneration (Orr et al., 2020). Understanding mechanisms of neuronal homeostasis in disease and injury are likely to provide insights into endogenous mechanisms with neuroprotective potential. Moreover, understanding pathological alterations in function are critical for shedding light on the timeline and mechanisms of neuronal loss and disease pathogenesis.

Glaucoma is a blinding neurodegenerative disease that strikes at retinal ganglion cells (RGCs), the output neurons of the retina (Calkins, 2012; Weinreb et al., 2014). It is commonly associated with elevated eye pressure, which damages RGC axons at the optic nerve head and culminates in the degeneration of their axon projections to visual regions of the brain and apoptotic loss of cell bodies in the retina. However, glaucoma-associated changes to visual function are likely the result of more than RGC and axonal degeneration, as cell loss is preceded by numerous changes in the structure and function of RGC-associated circuits. Within the retina, for instance, pressure elevation upregulates Na^+^ channel expression and spiking behavior of RGCs, alters the size and complexity of their dendritic fields, changes receptive field properties, leads to changes in post-synaptic receptor composition, and alters the responses of upstream retinal circuits (Della Santina et al., 2013; Frankfort et al., 2013; Wang et al., 2014; Pang et al., 2015; Ou et al., 2016; Bhandari et al., 2019; McGrady et al., 2020; Sladek and Nawy, 2020). Within the optic projection and at RGC output sites in the brain, eye pressure and glaucomatous injury lead to alterations in optic nerve glial function and energy demands (Baltan et al., 2010; Inman and Harun-Or-Rashid, 2017; Jassim et al., 2019; Cooper et al., 2020), RGC axon terminal swelling and atrophy (Smith et al., 2016), changes in axon terminal mitochondrial health (Smith et al., 2016), altered synaptic vesicle release properties (Bhandari et al., 2019), and post-synaptic neuronal dendritic remodeling and somatic atrophy (Gupta and Yücel, 2003; Gupta et al., 2007, 2009; Liu et al., 2011, 2014; Bhandari et al., 2019). Such changes likely represent a mix of both homeostatic attempts at preserving retinal output fidelity and pathological alterations in neural function in glaucoma. Overall, the relative timing, balance, and interplay of these two possible processes remain unknown.

The goal of this study was to test the hypothesis that glaucoma progression triggers changes in neuronal and synaptic function in the dorsal lateral geniculate nucleus (dLGN), a major RGC projection target in the thalamus that underlies conscious, image-forming vision by receiving and processing RGC signals and relaying them to the primary visual cortex (Kerschensteiner and Guido, 2017; Seabrook et al., 2017). Long considered a simple relay structure, recent evidence has highlighted how changes in visual activity can alter dLGN synapses and neuronal function (Rose and Bonhoeffer, 2018). For instance, the maturation of neuronal excitability, dendritic structure, and synaptic transmission is regulated by visual experience and retinal inputs during development and young adulthood (Hooks and Chen, 2006; Hong and Chen, 2011; Seabrook et al., 2013; Louros et al., 2014; El-Danaf et al., 2015; Liang and Chen, 2020). Moreover, several studies have documented examples of functional alterations occurring in dLGN neurons and synapses in response to altered sensory input and in cases of disease and injury (Krahe and Guido, 2011; Araújo et al., 2017; Sommeijer et al., 2017; Rose and Bonhoeffer, 2018; Bhandari et al., 2019). This raises the possibility that RGC injury and dysfunction occurring in glaucoma can trigger compensatory homeostatic plasticity in dLGN synapses and neurons.

Therefore, we set out to determine whether and how changes to neuronal function relate to eye pressure and glaucomatous RGC degeneration to shed light on the links between eye pressure, neuronal homeostasis, and dysfunction in glaucoma. This was accomplished by using two complementary mouse models of glaucoma and probing for structural and functional alterations to neurons and synapses in the dLGN. Ultimately, we find changes in excitatory synaptic transmission onto the principal dLGN relay neurons. Additionally, glaucoma enhances its excitability in a manner associated with eye pressure in old mice. In 9-month-old D2 mice, we find RGC synaptic terminal loss associated with IOP and atrophy of neuronal cell bodies in the dLGN, which are likely to be signs of glaucomatous pathology. This implies that the dLGN in glaucoma is characterized by potentially overlapping processes of homeostatic modulation of function as well as alterations and degeneration that are linked to disease severity.

## Materials and Methods

### Animals

Animal protocols were approved by the Institutional Animal Care and Use Committee at the University of Nebraska Medical Center. DBA/2J (D2; Jax# 000671) were used as an inherited model of glaucoma (John et al., 1998; Libby et al., 2005; Howell et al., 2007) and bred in-house or purchased from Jackson Labs. A strain-matched control line that contains a wild-type allele of the *Gpnmb* gene and does not develop elevated eye pressure or glaucoma was used as a control (DBA/2J^*Gpnmb+*/SjJ^; D2-control; Jax# 007048; Howell et al., 2007). For microbead injection experiments, we used mice produced as a cross of Opn4^Cre/Cre^ (Ecker et al., 2010) and Ai32 (Jax# 024109; Madisen et al., 2012) lines (Opn4^Cre^; Ai32; Bhandari et al., 2019). Mice were housed in a 12/12 h light/dark cycle and provided with food and water *ad libitum*.

### Microbead Occlusion Model

To induce ocular hypertension, fluorescently-tagged polystyrene microspheres (10 microns, Invitrogen F8836) were bilaterally injected into the anterior chambers of Opn4^Cre^; Ai32 mice at ~6–8 weeks of age (Sappington et al., 2010; Calkins et al., 2018; Bhandari et al., 2019). This procedure was performed under isoflurane anesthesia and following the instillation of anesthetic eye drops (0.5% proparacaine, Akorn, Lake Forest, IL, USA). Pupils were dilated with 1% tropicamide eye drops. In total, a small volume of beads (~1–2 μl) at a concentration of ~14 × 10^6^ beads/ml was injected using a glass micropipette.

### Intraocular Pressure Measurements

Intraocular pressure (IOP) was monitored using a Tonolab rebound tonometer (iCare, Vantaa, Finland) in mice lightly anesthetized with isoflurane, as we have described previously (Bhandari et al., 2019). IOP was measured approximately monthly in D2 and D2-control mice beginning around 2–4 months of age and were measured before and at 2 days, 1, 2, and 4 weeks post-injection for microbead-injected mice. For assessing the cumulative injury effects of IOP in D2 mice, we calculated a “3-month cumulative IOP,” which for a given mouse, is the sum of monthly IOP measurements taken over a 3 month-span (IOP measurements at 7, 8, and 9 months of age) and averaged across the two eyes. For microbead injected mice, we calculated a cumulative ΔIOP over baseline for each mouse as the average across the two eyes of 1-, 2-, and 4-week post-injection IOP measurements over the pre-injection baseline.

### Brain Slice Patch-Clamp Electrophysiology

Two-hundred and fifty-micron thick coronal brain slices containing the dLGN were acutely prepared using the “protected recovery” method (Ting et al., 2014, 2018), as we have described previously (Bhandari et al., 2019; Van Hook, 2020). Following euthanasia by CO_2_ asphyxiation and cervical dislocation, brains were submerged in a slush of artificial cerebrospinal fluid (aCSF) comprised of (in mM) 128 NaCl, 2.5 KCl, 1.25 NaH_2_PO_4_, 24 NaHCO_3_, 12.5 glucose, 2 CaCl_2_, and 2 MgSO_4_ and continuously bubbled with a mixture of 5% CO_2_ and 95% O_2_. Slices were prepared on a vibrating microtome (Leica VT1000S) and hemisected through the midline. Slices were then incubated for ~12 min at 33°C in an N-methyl-D-glucamine-based solution (in mM: 92 NMDG, 2.5 KCl, 1.25 NaH_2_PO_4_, 25 glucose, 30 NaHCO_3_, 20 HEPES, 0.5 CaCl_2_, 10 MgSO_4_, 2 thiourea, 5 L-ascorbic acid, and 3 Na-pyruvate), after which they were transferred to a solution of room-temperature aCSF and allowed to recover for >1 h before beginning recording.

dLGN slices were transferred to a recording chamber on a fixed-stage upright microscope (Olympus BX51-WI) and superfused with aCSF at a rate of 2–4 ml/min. For recordings from D2 and D2-control mice, the aCSF was warmed to 30–33°C using an inline heater. For recordings from microbead-injected mice, experiments were performed at room temperature (~23°C) and the aCSF was supplemented with 60 μM picrotoxin. Thalamocortical relay (TC) neurons located in the dLGN core (>100 microns from the dorsolateral surface of the dLGN) were targeted for whole-cell recording based on soma size and shape and distinguished from interneurons by the presence of a pronounced low-voltage activated T-type Ca^2+^ current. For voltage-clamp experiments, the patch pipette solution was comprised of (in mM) 120 Cs-methanesulfonate, 2 EGTA, 10 HEPES, 8 TEA-Cl, 5 ATP-Mg, 0.5 GTP-Na_2_, 5 phosphocreatine-Na_2_, 2 QX-314 (*pH* = 7.4, 275 mOsm) while for current-clamp experiments, the pipette solution was comprised of (in mM) 120 K-gluconate, 8 KCl, 2 EGTA, 10 HEPES, 5 ATP-Mg, 0.5 GTP-Na_2_, 5 phosphocreatine (*pH* = 7.4, 275 mOsm). Miniature excitatory postsynaptic currents (mEPSCs) were recorded in the absence of stimulation at a holding potential of −70 mV. TC neuron spiking was evoked using a series of depolarizing current injections (+40 to +560 pA, 500 ms) while membrane properties were monitored using hyperpolarizing injections (−20 to −100, 500 ms).

### Electrophysiology Analysis

Action potentials from current-clamp experiments were detected and counted using the “event detection” function of Clampfit. Input resistance (*R*_in_) was measured as the slope of a straight line fit to the voltage deflection amplitudes evoked by current stimuli of −20 and −40 pA. Membrane time constant (τ_m_) was measured with a single exponential fit to the voltage deflection evoked by a −20 pA step and was used along with *R*_in_ to calculate the membrane capacitance (*C*_m_ = τ_m_/*R*_in_). mEPSCs were detected and analyzed using MiniAnalysis software (Synaptosoft, Fort Lee, NJ, USA). For each cell, the first ~100 detected events were analyzed. All reported voltages are corrected for a measured −14 mV liquid junction potential for the K-based pipette solution and a −10 mV liquid junction potential for the Cs-based solution.

### Immunofluorescence Staining

For labeling RGC somata, we performed immunofluorescence staining of flat-mount retinas with a guinea pig-anti-RBPMS antibody (PhosphoSolutions, 1832-RBPMS, 1:500, RRID: AB_2492226; Rodriguez et al., 2014). Position in the retina was determined by staining with a primary antibody sensitive to S-opsin (rabbit-anti-s-opsin, 1:500, Millipore ABN1660; Sondereker et al., 2018; Stabio et al., 2018), which is expressed in a gradient along the dorsal-ventral axis of the retina (Applebury et al., 2000). Retinas were dissected into oxygenated aCSF or Ames solution. Four relieving cuts were made, and the retina was mounted on a nitrocellulose membrane (type AAWB, 0.8-micron pore size, Millipore, Burlington, MA, USA) and fixed by immersion in 4% paraformaldehyde for 30 min. Retinas were washed, blocked, and permeabilized in a solution of 1% Triton X-100, 0.5% DMSO, 5.5% donkey serum, and 5.5% goat serum for 1 h before being incubated overnight at 4°C in the same solution plus the addition of the primary antibodies. Retinas were then washed 6×, blocked/permeabilized again, and incubated with an AlexaFluor 568-conjugated goat-anti-guinea pig and an AlexaFluor 488-conjugated donkey-anti-rabbit secondary antibodies for 2 h at room temperature. After washing 3× in PBS, retinas were removed from the nitrocellulose membranes, mounted on Superfrost Plus slides, and coverslipped with VectaShield HardSet.

For immunofluorescence staining of dLGN sections, mice were euthanized and brains dissected, as described above. After a brief rinse in PBS, they were immersed in 4% paraformaldehyde for 4 h after which they were rinsed 3× in PBS and cryoprotected for one to three nights by immersion in 30% sucrose in PBS at 4°C. Brains were embedded in 3% agar, cut into 50-micron sections with a Leica VT1000S vibratome, mounted on SuperFrost Plus slides, and stored at −20°C. After blocking/permeabilization in PBS containing 0.5% Triton X-100, 5.5% (donkey and/or goat serum), slices were stained using either a rabbit-anti-vGlut2 primary antibody (1:250, Cedarlane/Synaptic Systems #135403, RRID: AB_887883), a guinea pig-anti-vGlut1 antibody (1:500, EMD Millipore AB5905, RRID: AB_2301751) or a combination of guinea pig-anti-NeuN polyclonal antibody (Millipore ABN90, RRID: AB_11205592, 1:500) and rabbit-anti-GAD65/67 polyclonal antibody (1:500, Millipore G5163, RRID: AB_477019) overnight at 4°C in a humidified chamber. For staining with the guinea pig primary antibody, we used a blocking solution containing both goat and donkey serum. Secondary antibodies were goat- or donkey-raised AlexaFluor 488 or 568-conjugated antibodies and were used at a concentration of 1:200. Slides were then washed 3× in PBS followed by 1× in dH_2_O and coverslipped using VectaShield HardSet.

### Imaging and Image Analysis

Imaging was performed with a 2-photon microscope with a 20× water-immersion objective (Scientifica) with the laser tuned to 800 nm. vGlut2 and vGlut1 images were acquired in frames of 185 × 185 μm (5.54 pixels/μm) with 0.5 μm *z*-axis spacing from the dLGN core. Four images per focal plane were averaged and a maximum intensity projection was created from five planes to give an effective *z*-slice thickness of 2.5 microns. The signal was automatically thresholded and vGlut2 and vGlut1 puncta were detected using the Synapse Counter plug-in in ImageJ (Dzyubenko et al., 2016) with a size threshold of 9 μm^2^ for vGlut2 and 0.25 μm^2^ for vGlut1.

For imaging RGCs, RBPMS-stained retinas were imaged in frames of 350 × 350 μm (1.04 pixels/μm). A series of images were acquired through the ganglion cell layer at 1-micron spacing in four quadrants of the central and peripheral retina (~500 and 1,700 μm from the optic nerve head, respectively). Each of the four quadrants was identified as temporal, nasal, ventral, or dorsal based on the dorsal-ventral gradient of s-opsin cone labeling (Applebury et al., 2000). Four images per focal plane were averaged for analysis and RGCs were counted using the Cell Counter plug-in in ImageJ.

NeuN and GAD65/67-stained neurons in the dLGN core were imaged in frames of 185 × 185 μm (5.54 pixels/μm) with 1-micron *z*-axis spacing. Four images per plane were acquired and averaged for analysis. To measure cross-sectional soma area, NeuN-stained cell bodies were traced by hand in the image plane of each cells’ greatest area. After tracing, the regions of interest were superimposed on the GAD65/67 signal, and GAD65/67-positive cell bodies were excluded from soma area analysis.

### Statistical Analysis

Data are presented as mean ± SEM unless indicated otherwise. Statistical significance was assessed using several different approaches. A two-tailed nested *t*-test was performed using GraphPad Prism 8 for electrophysiology experiments in which multiple cells were recorded from multiple animals, as indicated below. Otherwise, an unpaired two-tailed Student’s *t*-test was used, as indicated. The significance threshold was set at *p* < 0.05 for *t*-tests. Linear regression was used to test for correlations of mEPSC, excitability, RGC density, and vGlut2 staining with intraocular pressure. For correlation analysis, the mean values of each parameter for each mouse were used for the fit. A *p*-value <0.05 was considered a statistically significant correlation. To test for age effects and interactions of age and genotype, a two-way mixed ANOVA with a significance threshold of *p* < 0.05 was performed in SPSS (IBM, Armonk, NY, USA) with mouse age (4 and 9 m) and genotype (D2 and D2-control) independent variables and various cellular/synaptic parameters used as dependent variables.

## Results

### dLGN TC Neuron Excitability Is Enhanced in D2 Mice

**Figure 1:**
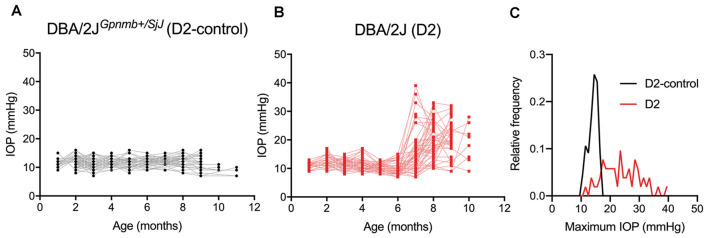
Eye pressure is elevated in DBA/2J mice. **(A,B)** Intraocular pressure (IOP) measurements from DBA/2J^*Gpnmb+/SjJ*^ (D2-control, **A**) mice (*n* = 62 eyes from 31 mice) and DBA/2J (D2, **B**) mice (*n* = 66 eyes from 33 mice). **(C)** Histogram of peak IOP values obtained from D2 and D2-control mice. D2-control IOPs were narrowly distributed (median = 14 mmHg) while the peak IOPs from D2 mice showed a broader distribution (median = 23 mmHg).

To determine whether glaucoma and IOP affect the function of neurons in the dLGN, we made use of the DBA/2J (D2) line of mice. These mice displayed elevated IOP beginning around 7 months of age when compared to a strain-matched control mouse line (DBA/2J^Gpnmb+/SjJ^; D2-control; Figure 1). The IOP elevation was variable in D2 mice and peak IOP within the population (aged 9–10 months) ranged from 11 to 39 mmHg (*n* = 52 eyes, 26 mice). This distribution was significantly different than the maximum IOP values from D2-control mice (*p* < 0.00001, K–S test). At 9 months of age, for instance, IOP was 20.2 ± 6.2 mmHg (Mean ± SD; *n* = 32 eyes, 16 mice) for D2 compared to 11.9 ± 2.4 for 9-month-old D2-control mice (*n* = 16 eyes, eight mice; *p* < 0.00001, unpaired *t*-test). This timeline and variability in IOP are consistent with prior studies of D2 mice (John et al., 1998; Libby et al., 2005; Inman et al., 2006).

**Figure 2:**
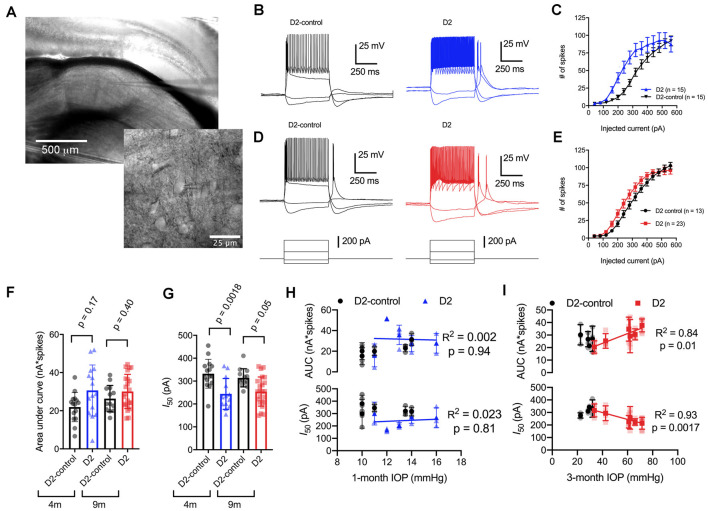
Enhancement of thalamocortical (TC) neuron spiking in D2 mice. **(A)** Left, microscopy image of dorsal lateral geniculate nucleus (dLGN) with a patch-clamp electrode in a coronal brain slice. Right, higher magnification image showing a TC neuron with the patch-clamp electrode. **(B)** Evoked spiking responses to depolarizing current injection (+120 and + 320 pA) and voltage deflections to hyperpolarizing current injections (−20 and −80 pA) in 4 m D2 mice (15 cells, five mice) and D2-control mice (*n* = 15 cells, five mice). **(C)** Plot of current injection and counts of evoked spikes (mean ± SEM) show that TC neurons from 4 m D2 mice fired more spikes (unpaired *t*-test). **(D)** Spiking and hyperpolarizing voltage responses from 9 m D2-control and D2 mice. **(E)** Similar to panel **(C)**, TC neurons from 9 m D2 mice (*n* = 23 cells, six mice) were slightly more excitable than from D2-control mice (*n* = 13 cells, four mice). **(F)** Mean ± SD of the area under the curve of spiking responses and data points from individual cells from 4 to 9 m D2 and D2-control mice. Significance was assessed using a nested *t*-test approach. **(G)** Mean ± SD and individual cell data points of the half-maximal current injection from a Boltzmann fit to the current-spiking relationships. **(H)** There was no significant correlation of area under the curve (AUC) or *I*_50_ with IOP (measured at 4 m of age) as assessed with linear regression for 4 m D2 mice. Regression was performed on mean values of AUC and *I*_50_ for each mouse rather than on individual cells. **(I)** In 9 m D2 mice, there was a significant correlation of AUC and *I*_50_ with a 3-month cumulative IOP averaged across two eyes for each mouse.

Glaucoma is triggered by an injury to retinal ganglion cell axons, which comprise the optic nerve and are responsible for carrying information to visual centers of the brain. Because of this, we next sought to determine whether D2 mice display changes in the function of neurons in the dLGN, a key RGC projection target for conscious vision, and relate those changes to IOP. To do this, we targeted thalamocortical (TC) relay neurons in the dLGN for whole-cell current-clamp recording in acute coronal dLGN brain slices (Figure 2). Experiments were performed in slices from D2 and D2-control mice at 4 and 9 months (4 and 9 m) of age to compare function at an earlier (pre-ocular hypertension/pre-OHT) and a later (OHT) time point.

When we used depolarizing current injections (500 ms, 40–560 pA; Figures 2B–E), we found that spiking was enhanced in 4-month-old (4 m) D2 mice compared to D2-controls. For instance, a 240 pA current injection evoked 19 ± 4 action potentials in D2-control mice (*n* = 15 cells, five mice) whereas TC neurons from age-matched D2 mice fired 55 ± 9 action potentials (*n* = 15 cells, five mice; *p* = 0.0016). We further quantified the enhanced excitability by measuring the area under the curve (AUC) of the current-spike relationship as well as the half-maximal current stimulus of a Boltzmann fit (*I*_50_; Figures 2F,G). In this analysis, the AUC from 4 m D2 cells was (31 ± 3 nA*spikes) compared to 4 m D2 controls (22 ± 2 nA*spikes). This difference was not statistically significant (*p* = 0.17). The *I*_50_ was significantly shifted left, from 332 ± 16 pA for D2-controls to 244 ± 17 pA for D2 mice (*p* = 0.0018).

When we performed similar experiments with 9 m D2 and D2-control mice, excitability was similar in TC neurons from D2 and D2-controls. For instance, the AUC was 26 ± 2 nA*spikes in 9 m D2-control mice (*n* = 13 cells, four mice) and 30 ± 2 nA*spikes in 9 m D2 mice (*n* = 23 cells, six mice; *p* = 0.4). Additionally, the *I*_50_ was 314 ± 11 pA in D2-controls and 254 ± 13 in D2’s (*p* = 0.048). When we compared age, genotype, and excitability parameters using a two-way mixed ANOVA, there was a significant effect of genotype on *I*_50_ (*p* < 0.001) and AUC (*p* = 0.012), but not of age (*p* = 0.80 and *p* = 0.42, respectively). There was also no significant interaction of age and genotype on either parameter (*I*_50_, *p* = 0.37; AUC, *p* = 0.31).

Notably, we did find that there was considerable variability of the spiking in 9 m D2 TC neurons relative to D2-controls. For instance, the SD of the *I*_50_ was 64 pA for 9 m D2 and 39 pA for D2-control while for AUC, the SD was 9.0 for D2 and 6.9 for D2-control. As shown above, there was also wide variability in D2 mouse IOP values at 9 months. We turned this to our advantage by testing whether AUC and *I*_50_ were related to the cumulative IOP experienced by the D2 mouse visual system by plotting AUC and *I*_50_ against the 3-month cumulative IOP (Figures 2H,I). When we did this, we found that the AUC positively correlated with cumulative IOP (*p* = 0.01) while the *I*_50_ negatively correlated with cumulative IOP (*p* = 0.002). In contrast, IOP was not elevated in the 4-month-old D2 mice and there was no significant correlation of AUC or *I*_50_ with IOP (*p* > 0.05). Thus, within the population of 9 m D2 mice, higher eye pressure was associated with greater dLGN TC neuron excitability.

**Figure 3:**
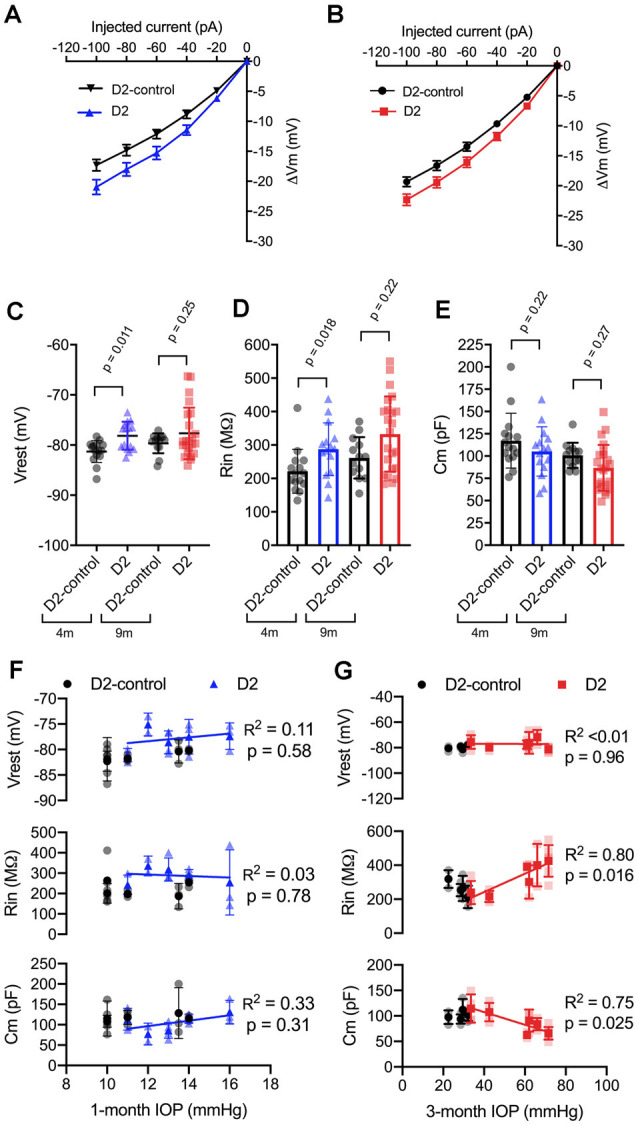
Changes to passive membrane properties support enhanced TC neuron excitability in D2 mice. **(A,B)** Plots of voltage deflection evoked by hyperpolarizing current injections in 4 m **(A)** and 9 m **(B)** D2-control and D2 mice. In D2 mice, hyperpolarizing current injections evoked larger voltage changes (unpaired *t*-test). **(C)** Resting membrane potential (*V*_rest_) measured in D2 and D2-control mice at 9 and 4 m. *V*_rest_ was significantly depolarized in 4 m D2 mice, but not 9 m D2 mice when compared with age-matched controls (nested *t*-test). **(D)** Input resistance (*R*_in_), measured using a linear fit to voltage deflections evoked by −20 and −40 pA, was significantly elevated in 4 m D2 TC neurons, but not for 9 m when compared using a nested *t*-test. **(E)** Membrane capacitance was not significantly different between D2-control and D2 mice at 4 m or 9 m (nested *t*-test). **(F)** There was no significant correlation of *V*_rest_, *R*_in_, or *C*_m_ with IOP at 4 m as assessed using linear regression. **(G)** Although there was no significant correlation of *V*_rest_ in 9 m D2 TC neurons with the 3-month cumulative IOP, *R*_in_, and *C*_m_ did correlate in a manner that supports enhanced TC neuron excitability in D2 mice with elevated IOP.

We next sought to probe the cellular mechanisms underlying this change in neuronal excitability (Figure 3) and found that the increase in TC neuron excitability in both 4 and 9 m D2 mice was associated with changes in membrane properties that support increased action potential firing. In 4 m mice, D2 TC neurons were slightly depolarized relative to D2-controls (D2: −78.2 ± 0.7 mV; D2-control: −81.3 ± 0.6 mV; *p* = 0.011), which brings their membrane potential closer to action potential threshold. Additionally, input resistance (*R*_in_), measured with a linear fit to voltage responses to hyperpolarizing current injections (Figures 3A,B), was higher in 4 m D2 mice (D2: 288 ± 20 MΩ; D2-control: 221 ± 17 MΩ; *p* = 0.018). Membrane capacitance (*C*_m_), an electrical measure of cell surface area, was not significantly different between 4 m D2 and D2-control mice (D2: 105.2 ± 7.1 pF; D2-control: 117.3 ± 8.0 pF; *p* = 0.27).

In 9 m D2 mice, there were no significant differences in *V*_rest_, *R*_in_, or *C*_m_ between D2 and D2-controls. For instance, *V*_rest_ was −77.7 ± 1 mV for 9 m D2 and −79.7 ± 0.6 mV for D2-controls (*p* = 0.25). *C*_m_ was 86.7 ± 5.4 pF in D2 s, which was not significantly different from measurements in D2-controls (100.8 ± 3.9 pF, *p* = 0.27). Likewise, *R*_in_ was not significantly different in 9 m D2 s (333 ± 23 MΩ) compared to 9 m D2-controls (262 ± 17 MΩ, *p* = 0.22). Using the two-way mixed ANOVA, there was a statistically significant effect of genotype on *V*_rest_ (*p* = 0.006) and *R*_in_ (*p* = 0.003) with no statistically significant effect of age (*V*_rest_, *p* = 0.24; *R*_in_, *p* = 0.056) or an interaction of age and genotype (*V*_rest_, *p* = 0.52; *R*_in_, *p* = 0.91). For *C*_m_, there was a significant effect of genotype (*p* = 0.048) and age (*p* = 0.009), but no statistically significant interaction was detected (*p* = 0.88).

As for AUC and *I*_50_, above, we noted that there was considerable variability in *V*_rest_, *R*_in_, and *C*_m_. Therefore, we tested whether there was a correlation of each parameter with IOP in D2 mice (Figures 3F,G). For mean values of cells recorded from 9 m D2 mice, *C*_m_ was negatively correlated with IOP (*p* = 0.025), while *R*_in_ was positively correlated with IOP (*p* = 0.011). In contrast, there was no significant correlation between *V*_rest_ and IOP (*p* = 0.96). Consistent with a role for *C*_m_ in influencing *R*_in_ in 9 m D2 TC neuron, *R*_in_ was negatively correlated with *C*_m_ assessed with a linear regression (*R^2^* = 0.73, *p* < 0.0001). Moreover, neither *C*_m_, *R*_in_, nor *V*_rest_ were significantly correlated with IOP in 4 m D2 mice (*p* = 0.31, *p* = 0.78, *p* = 0.58, respectively). Likewise, *C*_m_ and *R*_in_ were not significantly correlated in 4 m D2 mice (*R^2^* = 0.25, *p* = 0.06). Thus, in 9 m D2 mice, there was an association of several TC neuron parameters with the cumulative IOP such that cells from mice with higher IOP were more excitable, had a higher *R*_in_, and had a lower *C*_m_.

### TC Neuron Soma Size Is Reduced in Aged D2 Mice

**Figure 4:**
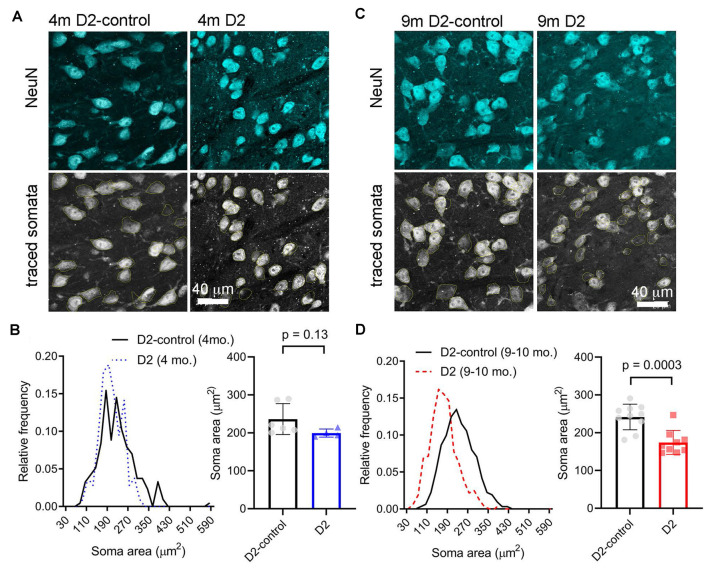
Measurement of TC neuron soma size in 4 to 9 m D2 mice. Fifty-micron dLGN sections were stained using a NeuN primary antibody to label neurons while a GAD65/67 antibody was used to identify and exclude GABAergic interneurons. **(A,C)** After imaging, TC neuron somata were traced. Lower panels show the somatic outlines (yellow). **(B)** At 4 m, TC neurons size was not significantly different between D2 mice (*n* = 106 cells from four mice), and D2-controls (*n* = 214 cells from six mice, *p* = 0.13, nested *t*-test). **(D)** At 9 m, TC neuron soma size was reduced in D2 mice (*n* = 321 cells from nine mice) compared to D2-controls (*n* = 312 cells from 10 mice; *p* = 0.0003, nested *t*-test).

A reduction in cell size can contribute to increased *R*_in_ and would be consistent with the detected decrease in *C*_m_. We next sought to verify this electrophysiological finding using a parallel anatomical approach in which we identified neuronal cell bodies in the dLGN using a NeuN antibody and measured their cross-sectional area in D2 and D2-control tissue from 4m- and 9m-old mice (Figure 4). GABAergic interneurons were identified by staining with a GAD65/67 antibody and were excluded to focus this analysis on the excitatory TC relay neurons in the dLGN. At 4 m, there was no significant difference in the TC neuron soma area. The median was 229 μm^2^ in D2 control (interquartile range, IQR: 189–274 μm^2^; *n* = 214 cells from six mice) and 196 μm^2^ in D2 mice (IQR: 170–234 μm^2^; *n* = 106 cells from four mice; *p* = 0.13, nested *t*-test). At 9 m, the difference was pronounced, with the median soma area being 233 μm^2^ in D2-controls (IQR: 196–280 μm^2^; *n* = 312 cells from 10 mice) and the median soma area being 167 μm^2^ in D2 mice (IQR: 135–199 μm^2^; *n* = 321 cells from nine mice; *p* = 0.0003, nested *t*-test). This anatomical approach supports electrophysiological findings that TC neuron size is reduced in the 9 m D2 dLGN and suggests that excitability changes at 9 m might be linked to TC neuron atrophy.

### Enhancement of TC Neuron Excitability in an Inducible Ocular Hypertension (OHT) Model

**Figure 5:**
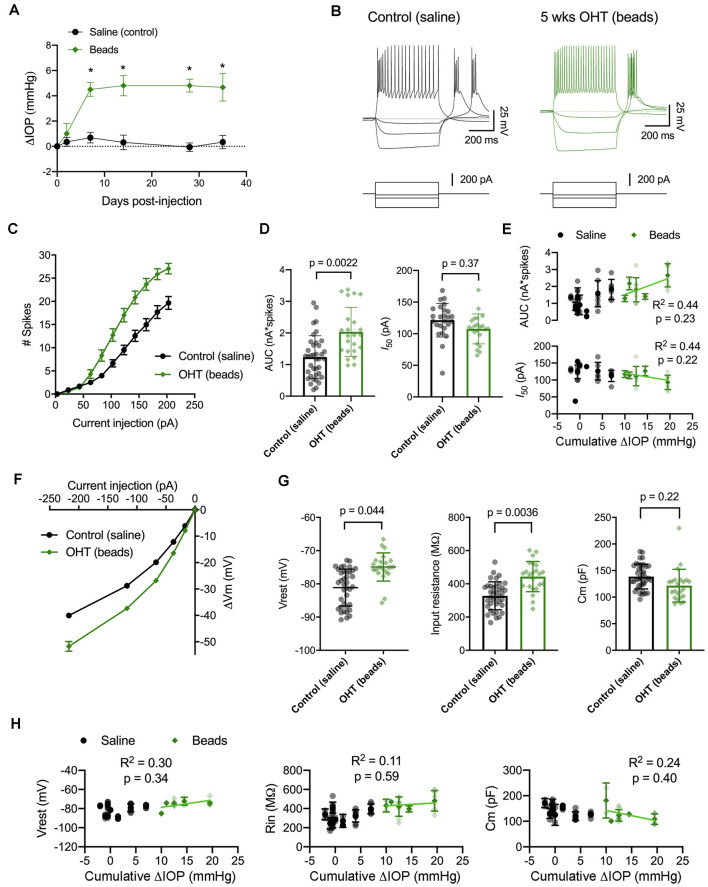
IOP elevation in the microbead model enhances dLGN TC neuron excitability. **(A)** Following bilateral anterior chamber injection of 10-micron polystyrene microbeads, IOP was elevated by ~5 mmHg (*n* = 10 eyes from five mice) compared to saline-injected control mice (*n* = 18 eyes from nine mice; *p < 0.05, unpaired *t*-test). **(B)** Whole-cell current-clamp recordings from TC neurons in dLGN coronal slices show spiking and hyperpolarizing voltage responses to depolarizing (+180 pA) and hyperpolarizing (−20, −70, and −220 pA) current injections in TC neurons from saline-and bead-injected mice. **(C)** Plot of mean ± SEM of current-evoked spiking (*p < 0.05, unpaired *t*-test). Saline data are from 38 cells from nine mice while microbead data are from 23 cells from five mice. **(D)** AUC was significantly elevated in cells from bead-injected mice compared to saline-injected controls (nested *t*-test). *I*_50_ was slightly reduced, although not significantly. Bars and error bars show mean ± SD while data points are measurements from individual cells. **(E)** There was no significant correlation of AUC or *I*_50_ with IOP from bead-injected mice as assessed with linear regression. Cumulative ΔIOP represents the between-eyes average of IOP measurements taken at 1, 2, and 4 weeks post-injection. **(F)** Voltage responses to hyperpolarizing current injections were used to measure passive membrane properties from the same population of cells. **(G)** TC neurons from bead-injected mice were slightly depolarized relative to saline-injected controls (nested *t*-test). *R*_in_ was significantly elevated and *C*_m_ was not significantly different. **(H)** Passive membrane properties did not significantly correlate with IOP as assessed with linear regression.

To test whether the increase in dLGN TC neuron excitability is a common feature of experimental glaucoma or instead of a quirk of the D2 mouse, we next turned to an inducible model in which eye pressure is elevated by injection of microbeads into the anterior chamber (Sappington et al., 2010; Calkins et al., 2018; Bhandari et al., 2019). This IOP elevation led to an approximately 5 mmHg increase in IOP over baseline (~30% increase; Figure 5A), which we have shown previously to trigger changes in the function of retinogeniculate (RG) synapses and TC neuron dendritic structure with minimal RGC loss 5 weeks after injection (Bhandari et al., 2019).

Here, we performed current-clamp recordings to measure neuronal excitability and membrane properties from mice with microbead-induced OHT (Figure 5B), similar to experiments with D2 mice, above. One difference was that experiments with dLGN slices from bead-injected mice were recorded at room temperature rather than warmed, which changes the evoked spiking and membrane properties of TC neurons (Van Hook, 2020). Still, in bead-injected mice, we found that TC neuron excitability was enhanced when compared to TC neurons from saline-injected controls (Figures 5C–E). This was reflected as an increase in the number of action potentials evoked by depolarizing current injection (20–220 pA) and further demonstrated as an increase in the AUC, from 1.2 ± 0.1 nA*spikes (*n* = 39 cells, nine mice) to 2.0 ± 0.2 nA*spikes (*n* = 24 cells, four mice; *p* = 0.022). Although there was a trend toward a leftward shift in *I*_50_ (saline: 121 ± 5 pA; beads: 108 ± 5 pA), the difference was not statistically significant (*p* = 0.37). Additionally, there was no significant correlation of either AUC (*p* = 0.23) or *I*_50_ (*p* = 0.22) with IOP for bead-injected mice.

We next compared membrane properties, including *V*_rest_, *R*_in_, and *C*_m_, between TC neurons from bead- and saline-injected mice as for D2 and D2-control mice, above (Figures 5F–H). We found that *V*_rest_ was slightly depolarized in TC neurons from bead-injected mice when compared to controls (beads: −74.9 ± 0.6 mV, *n* = 24 cells, five mice; saline: −80.9 ± 0.8 mV, *n* = 39 cells, nine mice; *p* = 0.044). Additionally, *R*_in_ was elevated in bead-injected mice relative to saline-injected controls (beads: 443 ± 20 MΩ; saline: 328 ± 14 MΩ; *p* = 0.0036). There was a trend toward a reduced *C*_m_ (beads: 121 ± 4 pF; saline: 138 ± 5 pF), but the difference was not significant (*p* = 0.22). Also, similar to the 4 m D2 mice, there was no significant correlation of *V*_rest_ (*p* = 0.34), *R*_in_ (*p* = 0.59), or *C*_m_ (*p* = 0.40) with IOP in microbead-injected mice.

### Reduced mEPSC Frequency in D2 Mice

**Figure 6:**
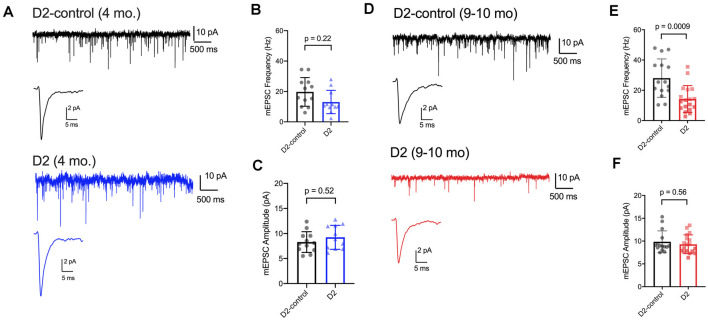
mEPSC frequency was reduced in D2 mice. **(A)** Whole-cell voltage-clamp records of mEPSCs recorded in the absence of stimulation for 4 m D2-control (*n* = 12 cells from five mice) and D2 mice (*n* = 11 cells from four mice). Single events are the average of events detected in each cell. **(B)** Mean ± SD of mEPSC frequency was slightly reduced in 4 m D2 mice. Individual points show the average instantaneous frequency for all events from individual cells. The difference was not statistically significant when assessed with a nested *t*-test. **(C)** mESPC amplitude was not different between 4 m D2-controls and D2 mice. **(D)** mEPSCs recorded from TC neurons from 9 m D2-control (*n* = 15 cells from four mice) and D2 mice (*n* = 18 cells from six mice). **(E)** mEPSC frequency was reduced at 9 m. **(F)** mEPSC amplitude was similar in D2-control and D2 TC neurons at 9 m.

We have previously shown that IOP elevation in the microbead model leads to a reduction in the frequency of miniature excitatory postsynaptic currents (mEPSCs) recorded from dLGN TC neurons (Bhandari et al., 2019). Therefore, we next used whole-cell voltage-clamp recordings of dLGN TC neurons from 4 to 9 m D2 mice to probe whether and how excitatory inputs are altered in these mice as well (Figure 6). For 4 m mice (Figures 6A–C), we found that mEPSC amplitude was similar in D2 and D2-control mice (D2-control: 8.3 ± 0.6 pA, *n* = 12 cells, five mice; D2: 9.2 ± 0.7 pA, *n* = 11 cells, four mice; *p* = 0.52). mEPSCs arising from cortical inputs are slower than those from RGC inputs due to their concentration at more distal dendritic sites and consequent dendritic filtering (Williams and Mitchell, 2008). Shifts in relative proportions of cortical-vs-retinal inputs can be reflected in changes in the mEPSC decay kinetics as occurs in monocular deprivation (Krahe and Guido, 2011). However, we did not observe any significant shift in mEPSC decay time constant in 4 m TC neurons (D2-control: 2.1 ± 0.2; D2: 2.3 ± 0.3 ms; *p* = 0.74). The mEPSC frequency at was not significantly different between 4 m D2 and D2-controls (D2-control: 19.7 ± 2.7 Hz; D2: 13.0 ± 2.3 Hz; *p* = 0.22, nested *t*-test).

In 9 m mice (Figures 6D–F), mEPSC amplitude was similar between D2 and D2-control (D2-control: 9.8 ± 0.6 pA, *n* = 15 cells, four mice; D2: 9.3 ± 0.5 pA, *n* = 18 cells, six mice; *p* = 0.56). Additionally, the mEPSC decay time constants were not significantly different (D2-control: 1.98 ± 0.09 ms; D2: 1.86 ± 0.11 ms; *p* = 0.84). mEPSC frequency was significantly lower in 9 m D2 mice compared to D2-controls (D2-control: 28 ± 3.3 Hz; D2: 14.3 ± 2.1 Hz; *p* = 0.0009). In contrast to our findings with excitability and membrane properties, there was no significant correlation of mEPSC frequency with the 3-month cumulative IOP (*R^2^* = 0.48; *p* = 0.13). Using the two-way mixed ANOVA, there was a significant effect of genotype on mEPSC frequency (*p* < 0.001), but no statistically significant effect of age (*p* = 0.084) or significant interaction of age and genotype (*p* = 0.20).

### Loss of RGC Axon Terminals in the dLGN of D2 Mice

Prior studies have indicated that RGC axon terminals in the superior colliculus (SC) are lost fairly late in disease in DBA/2J and microbead-injected mice and that terminal loss is preceded by swelling followed by atrophy (Crish et al., 2010; Smith et al., 2016). Additionally, we have shown that 5 weeks of a relatively modest and sustained OHT triggered by anterior chamber microbead injections does not have any effect on RGC axon terminal size or density in the dLGN (Bhandari et al., 2019).

**Figure 7:**
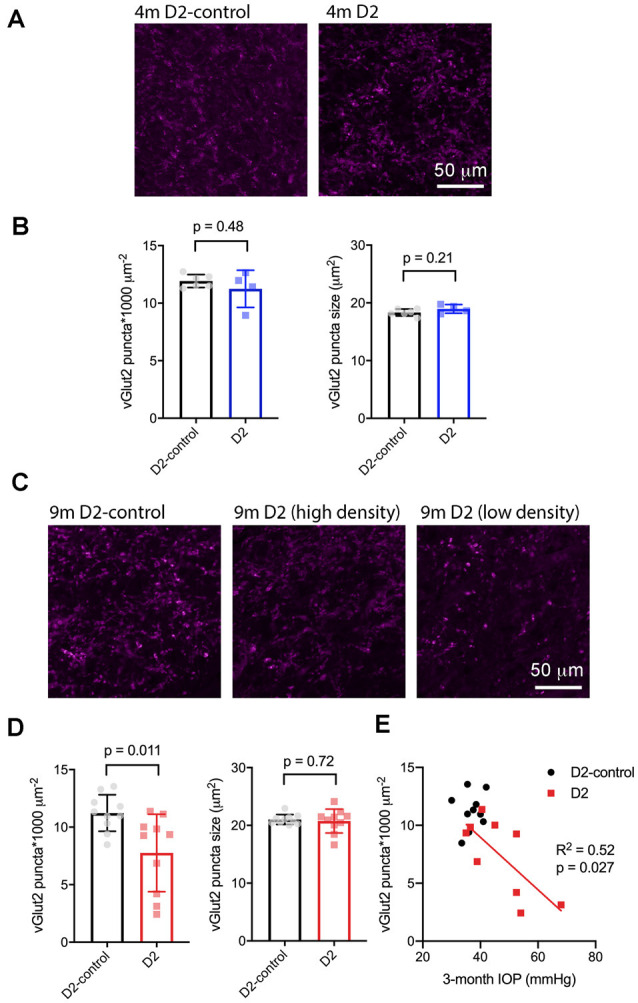
vGlut2-labeled RGC axon terminals are lost in an IOP-dependent manner in 9 m D2 mice. **(A)** 2.5-micron-thick maximum intensity projections of vGlut2 staining from the dLGN core of 4 m D2-control (*n* = six mice) and D2 mice (*n* = four mice). **(B)** Neither density nor size of detected vGlut2 puncta was significantly different between D2-control and D2 mice at 4 m. **(C)** vGlut2 images from 9 m D2-control (*n* = 10 mice) and D2 mice (*n* = 10 mice). For D2 mice, images are shown from mice with higher vGlut2 density (middle panel) and lower vGlut2 density (right panel) to illustrate the range of labeling seen. **(D)** vGlut2 density is significantly reduced in 9 m D2 mice compared to D2-controls (unpaired *t*-test) while vGlut2 puncta size is not different. **(E)** vGlut2 density in 9 m D2 mice dLGN sections significantly correlated with the 3-month cumulative IOP as assessed with linear regression.

Next, we sought to determine whether the size or density of RGC axon terminals, stained with an antibody raised against vGlut2, which is a selective label for RGC axon terminals in the dLGN, were altered in D2 mice at a young age (4 m) and in older mice (9 m) with elevated eye pressure (Figure 7). In sections from 4 m D2 mice (Figures 7A,B), we found that vGlut2 punctum density was similar to D2-control mice (D2: 11.2 ± 0.8 puncta/1,000 μm^2^, *n* = four mice; D2-control: 11.9 ± 0.2 puncta/1,000 μm^2^, *n* = six mice; *p* = 0.48). We found no evidence for RGC axon terminal atrophy or swelling, as punctum size was similar in controls and D2 (D2: 19.0 ± 0.4 μm^2^; D2-control; 18.3 ± 0.2 μm^2^, *p* = 0.21).

Similar to results at 4 months of age, there was no change in vGlut2 punctum size at 9 m (D2: 20.8 ± 0.44 μm^2^, *n* = 10; D2-control: 21 ± 0.3 μm^2^, *n* = 10; *p* = 0.72; Figures 7C,D). Although punctum size was unchanged, vGlut2 density was significantly reduced in D2 mice compared to controls (Figure 7D; D2: 7.8 ± 1.2 puncta/1,000 μm^2^; D2-control: 11.2 ± 0.5 puncta/1,000 μm^2^; *p* = 0.011). Notably, there was considerable variability in vGlut2 punctum density, with a range of 2.4–11.4 puncta/1,000 μm^2^ in D2 compared to 8.5–13.5 puncta/1,000 μm^2^ in controls. To test whether the diversity in vGlut2 density in D2 mice might be related to IOP, we tested for correlation of vGlut2 punctum density with the 3-month cumulative IOP (Figure 7E). Indeed, vGlut2 density was negatively correlated with the 3-month cumulative IOP (*p* = 0.027), suggesting that a greater loss of RGC axon terminals is associated with a higher cumulative IOP in 9 m D2 mice.

**Figure 8:**
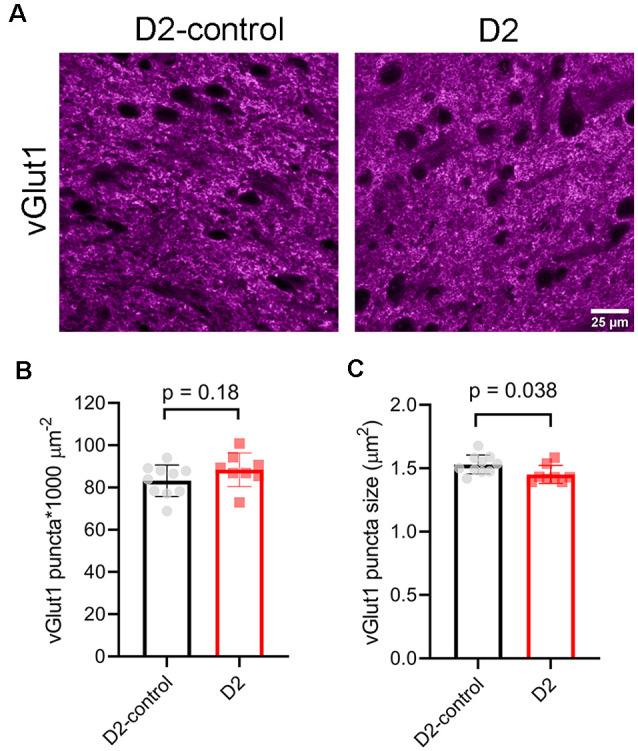
vGlut1-labeled corticothalamic axon terminals in 9 m D2 mice. **(A)** 2.5-micron-thick maximum intensity projections of vGlut1 staining from the dLGN core of 9 m D2-control and D2 mice. **(B)** The density of detected vGlut1 puncta (mean ± SD) was not significantly different between D2 (*n* = 8 mice) and D2-control (*n* = 10 mice, unpaired *t*-test). **(C)** vGlut1 puncta were slightly smaller in 9 m D2 mice (unpaired *t*-test).

To test for potential contribution of degeneration of inputs from visual cortex, we stained dLGN sections from 9 m D2 mice and D2-controls with an antibody sensitive to vGlut1 (Figure 8). The density of detected vGlut1 puncta was not significantly different between dLGN from D2 (88 ± 3 puncta*1,000 μm^−2^, *n* = 8 mice) and D2-control (83 ± 2 puncta*1,000 μm^−2^, *n* = 10 mice; *p* = 0.18, unpaired *t*-test). However, the vGlut1 puncta were slightly smaller in 9 m D2 mice (1.45 ± 0.02 μm^2^) than in D2-control (1.53 ± 0.02 μm^2^, *p* = 0.038, unpaired *t*-test). This suggests that cortical inputs are slightly atrophied in 9 m D2 mice, possibly indicating cortico-thalamic (C-T) synaptic dysfunction. In contrast to the vGlut2 density data, there was no significant correlation of vGlut1 punctum size with the 3-month cumulative IOP (*R^2^* = 2.1E-5, *p* = 0.99).

### Eye-Pressure-Associated RGC Loss in D2 Mice

**Figure 9:**
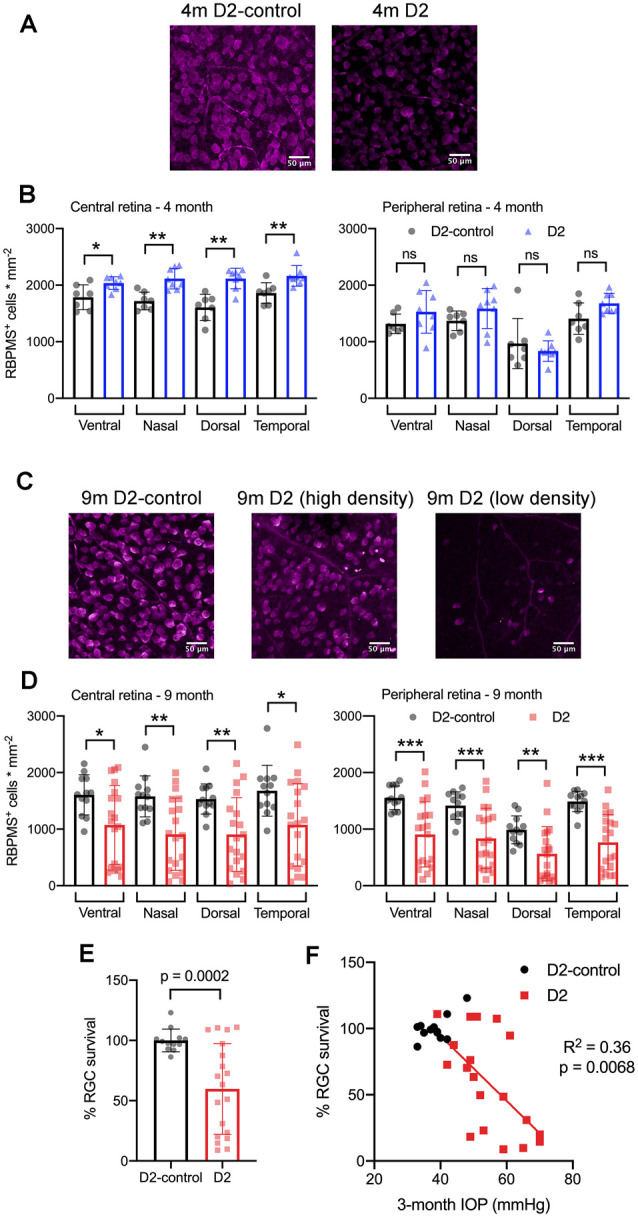
RBPMS-stained retinal ganglion cells are lost in an IOP-dependent manner in 9 m D2 mice. **(A)** Immunofluorescence images from RBPMS-stained retinas from a 4 m D2-control and a D2 mouse show similar RBPMS+ RGC soma density. **(B)** Quantification of RBPMS+ cell bodies in four quadrants of central and peripheral retina. Density was slightly higher in 4 m D2 mice (eight retinas from four mice) compared to D2-control mice (*n* = 7 retinas from four mice) in the central retina (*p < 0.05, unpaired *t*-test). Bars and error bars represent mean ± SD while individual points are RGC density measurements from individual retinas. **(C)** RBPMS images from 9 m D2-control (left) and D2 mouse retinas (center and right). Images from 9 m D2 retinas with higher and lower RBPMS+ cell density are shown to illustrate the diversity of RGC densities seen in D2 mice. **(D)** RBPMS+ cell density was lower in all four quadrants of central and peripheral retinas of 9 m D2 mice (*n* = 19 retinas from 12 mice) compared to D2-controls (*n* = 12 retinas from seven mice) illustrates the diversity of RGC survival from mouse-to-mouse. Bars and error bars represent mean ± SD while individual data points are RGC survival in individual retinas. **(E)** Quantification of RGC survival across the entire retina at 9 m. **(F)** RGC survival significantly correlated with the 3-month cumulative IOP in 9 m D2 mice, as assessed using linear regression. **p < 0.01; ***p < 0.005, ns: *p* > 0.05.

Although early changes to the structure and function of RGCs, their axons, and their projection targets in the brain are likely to contribute to visual impairment in glaucoma, RGC degeneration is thought to be the major cause of irreversible vision loss. Therefore, we sought to determine the differences in RGC degeneration in D2 mice at 4 and 9 m of age and relate RGC degeneration to IOP to have a marker of glaucoma severity as it relates to IOP, mouse age, and the functional changes to synapses and TC neuron spiking behavior in the dLGN. To accomplish this, we labeled RGCs by immunofluorescence staining for RBPMS, a selective RGC marker, and counted RGCs to determine their density in four quadrants of the central and peripheral retina (Figure 9).

At 4 m (Figures 9A,B), we found that there was no significant reduction in RGC density in D2 mice compared to D2-controls in either central or peripheral retina (D2: *n* = 8 retinas; D2-control: *n* = 7 retinas). Rather, in the central retina, the RGC density in D2 mice was slightly higher than RGC density in D2-controls. In the peripheral retina, however, there was no significant difference between D2- and D2-control RGC density in any of the four quadrants. At 9 m, however, RGC density was significantly lower in D2 than D2-controls (D2: *n* = 19 retinas; D2-control *n* = 12 retinas). This was the case in both the central retina and peripheral retina.

Across the retina, there was more variability in the RGC density in D2 mice compared to D2-controls at 9 m (Figures 9C–F). For instance, the standard deviation of RGC densities across four quadrants in central and peripheral retina in D2-control mice was 289 cells/mm^2^ while it was 599 cells/mm^2^ for age-matched D2-mice. We then tested whether the differences in RGC density were associated with IOP by calculating RGC survival as % of control RGC density and testing for correlation of RGC survival across the entire retina with the 3-month cumulative IOP. Indeed, RGC survival was negatively correlated with 3-month cumulative IOP (*p* = 0.0068), supporting a relationship between eye pressure and RGC loss in 9 m D2 mice.

## Discussion

In this study, we identify functional changes to the TC relay neurons and their synaptic inputs in the dLGN of DBA/2J mice, a widely-used and established rodent model of inherited glaucoma. Specifically, we find in younger D2 mice that do not have any detectable OHT or RGC loss, TC neurons more readily fire action potentials in response to depolarizing current stimuli and that this is associated with a slight depolarization and increase in neuronal input resistance. TC neuron excitability and membrane parameters were only subtly different from controls in 9-month D2 mice. However, there was considerable variability in neuronal excitability and passive membrane properties at 9 m, and these parameters correlated with D2 mouse IOP suggesting that within the population of 9 m D2 mice, TC neuron excitability is related to IOP. A two-way mixed ANOVA analysis did not reveal a significant effect of age on neuronal excitability parameters aside from *C*_m_ and consistently indicated that mouse genotype was determinative for increased TC neuron excitability. In mice with a modest OHT triggered by anterior chamber microbead injection, TC neuron excitability was also increased in a manner that resembled the 4 m D2 mouse population in that it was associated with a small depolarization and increase in *R*_in_ without significant change in *C*_m_. Moreover, excitability and passive membrane properties (AUC, *I*_50_, *C*_m_, *R*_in_) did not correlate with IOP in microbead-injected mice, similar to 4 m D2 mice.

We suggest that the pattern of functional changes in the dLGN might represent overlapping processes of homeostatic modulation of neuronal function combined with glaucomatous neurodegeneration and dysfunction (Turrigiano, 2011, 2012; Crish and Calkins, 2015). For instance, there was no detectable RGC loss in the 4 m D2 mice and IOP remained low, similar to D2-control mice. However, other studies have documented that functional changes can occur in the visual pathway of D2 mice at this age and earlier including the diminishment of optic nerve transport, remodeling of astrocytes in the optic nerve, and tau hyperphosphorylation (Crish et al., 2010; Dengler-Crish et al., 2014; Crish and Calkins, 2015; Cooper et al., 2016; Wilson et al., 2016). Thus, the enhanced excitability in 4 m D2 mice might represent functional homeostasis triggered as a downstream consequence of compromised optic nerve health and disrupted signal transmission from RGCs.

Indeed, homeostatic upregulation of intrinsic excitability has been documented in response to altered visual input in the visual cortex (Maffei and Turrigiano, 2008; Lambo and Turrigiano, 2013). OHT also alters RGC spike output, causing an early increase in excitability due to changed Na^+^ channel expression (Risner et al., 2018, 2020; McGrady et al., 2020). Other studies have documented diminishment of spontaneous and light-evoked RGC spike output occurring at different times with different IOP manipulations (Della Santina et al., 2013; Ou et al., 2016; Risner et al., 2018; Bhandari et al., 2019). Changes in RGC spike output might alter depolarization-triggered CREB activity in TC neurons (Pham et al., 2001; Guido, 2008; Krahe et al., 2012; Dilger et al., 2015). Altering RGC spiking might also influence the activity-dependent release of the neurotrophin BDNF, which is important in glaucoma pathology (Crish et al., 2013; Domenici et al., 2014; Gupta et al., 2014; Dekeyster et al., 2015; Valiente-Soriano et al., 2015), regulates the development of retinal ganglion cells and their brain projections (Marshak et al., 2007; Cohen-Cory et al., 2010; Nikolakopoulou et al., 2010), and is involved in homeostatic and experience-dependent plasticity of cortical neurons (Rutherford et al., 1998; Desai et al., 1999; Bracken and Turrigiano, 2009).

There was a notable correlation of neuronal excitability, including spiking parameters and passive membrane properties with IOP in 9 m mice. In these cases, higher IOP was associated with increased TC neuron excitability in the dLGN. Moreover, RGC density was significantly reduced across the retina in 9 m D2 mice and we found that higher IOP was also associated with lower RGC survival. This suggests that in the 9 m D2 population, changes in neuronal excitability are related to glaucoma severity. At this time point, we observed electrophysiological signatures of reduced TC neuron size and confirmed the TC neuron atrophy by measurements of TC neuron soma area. This is consistent with other studies of retinal projection targets in fairly late-stage glaucoma from human patients and primate and rodent animal studies which have also documented neuronal atrophy in the dLGN and SC (Yücel et al., 2001; Gupta et al., 2007, 2009). In this case, the increase in input resistance at 9 m is likely to be at least partially attributable to the reduced somatic area and this link is supported by the correlation of *C*_m_ and *R*_in_ in 9 m D2 TC neurons. Other factors such as the reduced synaptic inputs and the possibility of reduced neuronal membrane surface area due to dendritic pruning (Gupta et al., 2007; Bhandari et al., 2019) would contribute to this as well. At the 4 m time point and in microbead-injected mice, *V*_m_ was slightly depolarized, which supports neuronal excitability by bringing *V*_rest_ closer to the action potential threshold. *V*_rest_ is set by numerous cell-intrinsic and extrinsic mechanisms including leak and voltage-gated conductances and spontaneous synaptic inputs. GABAergic inhibition from both intrinsic interneurons and thalamic reticular nucleus projections acts on GABA_A_ ion channels and GABA_B_ metabotropic receptors on TC neurons and at RGC axon terminals (Chen and Regehr, 2003; Guido, 2018) and will also influence membrane resistance and resting potential. We did not explore whether the inhibitory transmission was altered in D2 mice of either age and such studies will be important for future work. Thus, in terms of neuronal excitability, we find a similar overall pattern of enhanced TC neuron firing in D2 mice of both ages compared to their age-matched controls with possible subtle differences in some of the potential underlying mechanisms. These patterns of functional changes might represent a shift from pre-degenerative homeostasis to pathological dysfunction associated with RGC somatic and axon terminal degeneration in D2 mice (Orr et al., 2020).

Probing synaptic function, we also found in 9 m D2 mice that the excitatory synaptic input onto dLGN TC neurons was reduced, as indicated by a reduction in mEPSC frequency. There was no change in mEPSC amplitude, suggesting that the postsynaptic AMPA receptor population was stable at excitatory synapses onto TC neurons. Moreover, in 9 m mice, there was a reduction in the density of vGlut2-labeled RGC axon terminals in the dLGN in a manner that correlated with IOP such that higher eye pressure was associated with a greater terminal loss.

Previous studies of the superior colliculus of D2 mice have reported that RGC axon terminals survive until fairly late in the disease and only degenerate well after RGC loss (Crish et al., 2010; Smith et al., 2016; Wilson et al., 2016). In our sample of 9 m D2 mice, we found that RGC axon terminals were lost in an IOP-dependent manner. We did not see evidence of either RGC axon terminal atrophy or swelling, as has been reported in ultrastructural studies of the SC (Smith et al., 2016). Several possibilities could account for this difference. First, it is possible that unlike in the SC, RGC terminals in the dLGN do not exhibit phases of swelling and atrophy before degeneration. This could represent a difference between these two visual structures, possibly resulting from the relative proximity of each structure to the retina, which is known to be associated with differential effects on the optic projection (Crish et al., 2010; Calkins, 2012). Alternatively, light microscopy as we implemented it might be too coarse of an imaging modality to detect subtle changes in RGC axon terminal structure if OHT triggers early swelling or later atrophy. Future ultrastructural studies of the dLGN would be able to address this possibility. Notably, Smith and colleagues combined their measurements of axon terminal size with anterograde transport studies and found that areas of the SC with intact transport from the retina tended to have terminal swelling while areas deficient in transport (taken as a sign of poorer health) tended to have atrophied terminals (Smith et al., 2016). We did not perform transport experiments as part of this study. Doing so in combination with vGlut2 staining in future studies might reveal a similar pattern in the dLGN.

Although retinogeniculate (RG) synapses provide the major excitatory drive for TC neurons, they comprise only ~10% of their total number of excitatory synaptic inputs (Bickford et al., 2010; Guido, 2018). The majority of excitatory inputs onto TC neurons are the result of corticothalamic (CT) synapses arising from layer VI of the visual cortex. These synapses function to modulate TC neuron responsiveness (Sherman and Guillery, 2002, 2011) and have a low release probability, as evidenced by their notable synaptic facilitation occurring during repeated stimulation (Turner and Salt, 1998; Jurgens et al., 2012). We find here that TC neurons from D2-control mice have a baseline mEPSC frequency of approximately 25 Hz, which is similar to what we have shown previously in recordings at ~30–33°C (Van Hook, 2020), although higher than the mEPSC frequency detected in recordings at room temperature (Bhandari et al., 2019; Van Hook, 2020). In the current study, we found that mEPSC frequency recorded in TC neurons was reduced in both 4 and 9 m D2 mice compared to their age-matched controls. The mechanisms underlying the reduced mEPSC frequency are unclear. IOP was not elevated in our sample of 4 m D2 mice, nor was mEPSC frequency correlated with IOP in 9 m D2 mice, in contrast to electrophysiological markers of TC neuron excitability, vGlut2 loss, and RGC loss. RGC axonal transport, cytoskeleton, and energy homeostasis are disrupted at fairly young ages in D2 mice, and this is accompanied by morphological changes in RGC axon terminal mitochondria in the SC (Crish et al., 2010; Smith et al., 2016). Synaptic transmission requires ample energy supply and functioning mitochondria to handle vesicle recycling, regulation of presynaptic vesicle pool size and release probability, and presynaptic Ca^2+^ handling (Ly and Verstreken, 2006). Therefore, perturbations of RGC axon terminal mitochondria and axonal energy supply are likely to influence synaptic output and might contribute to our observed synaptic changes.

In a previous study using the microbead approach to induce OHT, we suggested that a similar reduction in mEPSC frequency might be attributable to a loss of post-synaptic TC neuron synapses, as evidenced by a reduction in TC neuron dendritic complexity (Bhandari et al., 2019). Dendritic atrophy in retinorecipient neurons in late-stage glaucoma has also been documented elsewhere, yet we have not yet tested whether or along what time course this occurs in D2 mice.

Alternatively, changes in mEPSC frequency might arise from changes to non-RGC inputs. Krahe and Guido (2011) have shown that monocular deprivation leads to a homeostatic increase in synaptic input arising from the CT pathway in the dLGN. They reached this conclusion from observing changes in short-term plasticity during CT tract stimulation and an increase in the proportion of detected mEPSCs with slower decay kinetics, which results from filtering of signals arising at distal dendritic sites (Williams and Mitchell, 2008). It remains to be seen what proportion of TC neuron mEPSCs arise from retinal vs. cortical sources and whether OHT and optic nerve pathology can influence CT synaptic function. CT innervation is shaped by RG inputs during development (Seabrook et al., 2013) and dysfunction and injury to RGC axons might re-awaken those developmental processes in adulthood (Nahmani and Turrigiano, 2014). However, we did not detect any changes in mEPSC kinetics at either 4 m or 9 m, arguing against up-or down-regulation of CT input. Given the loss of vGlut2-labeled RGC axon terminals in 9 m D2 mice, it is likely that some reduction in mEPSC frequency is the result of less RGC input to each TC neuron at that time point. We did not see any loss of CT synaptic terminals in 9 m D2 mice (labeled *via* vGlut1 immunostaining, Figure 8), although they were slightly smaller, supporting the possibility that those terminals undergo some structural change that might either reflect or cause synaptic dysfunction.

It will ultimately take an examination of the properties of CT and RG synaptic function resulting from optic tract or CT tract stimulation to test for relative changes in synaptic function in D2 mice. We previously used optogenetic activation of RGC axons in microbead-injected mice to show that presynaptic vesicle release probability at RG synapses was increased in OHT (Bhandari et al., 2019). This was before substantial RGC degeneration or any detectable loss of vGlut2 staining in the dLGN, suggesting it might be an early, homeostatic response to pressure elevation. Numerous RGCs provide convergent synaptic input to each TC neuron, although only ~3 of those inputs are responsible for the majority of excitatory drive to each cell (Chen and Regehr, 2000; Hammer et al., 2015; Litvina and Chen, 2017; Rompani et al., 2017). Given the IOP-associated decline in vGlut2 staining in 9 m D2 mice, we do predict that later stages of the disease will be characterized by a reduction in total RG synaptic strength, possibly as a reduction in RGC convergence to each TC neuron, although the strength of inputs from single fibers is likely to be diminished as well as the axonal and synaptic function becomes more compromised.

In conclusion, prior studies have documented numerous changes to the structure of RGC axon terminals as well as to post-synaptic neurons in visual structures in the brain, especially in the SC, at various time points in glaucoma. The findings in the current study shed light on the progression of functional changes at the cellular and synaptic scale in the dLGN and relate those changes to established markers of glaucomatous progression—namely IOP and RGC loss in the retina. Specifically, we find that TC neuron excitability is enhanced in D2 mice and mice with experimentally-induced ocular hypertension. Moreover, changes to dLGN function appear to be related to some markers of disease progression, with RGC degeneration, loss of RGC axon terminals, and TC neuron excitability changes being correlated with eye pressure. This study enhances the picture of disease progression and provides important information linking the IOP to vision loss in glaucoma. Future work will need to examine other features of synaptic transmission such as corticothalamic inputs and GABAergic inhibition in D2 mice. A finer-grained examination of the timing of dLGN functional changes (i.e., very early following pressure elevation but before any RGC degeneration) will help clarify the overlap of homeostasis and pathology. Finally, future studies exploring the mechanistic link(s) between eye pressure and altered neuronal function in the dLGN will be essential for understanding the processes underlying glaucoma progression and vision loss.

## Data Availability Statement

The original contributions presented in the study are included in the article, further inquiries can be directed to the corresponding author.

## Ethics Statement

The animal study was reviewed and approved by the Institutional Animal Care and Use Committee at the University of Nebraska Medical Center.

## Author Contributions

MV: funding, experimental design, conducting experiments, analyzing data, and writing the manuscript. CM and JS: conducting experiments and analyzing data. EB: analyzing data. All authors contributed to the article and approved the submitted version.

## Conflict of Interest

The authors declare that the research was conducted in the absence of any commercial or financial relationships that could be construed as a potential conflict of interest.
